# Farmers’ pesticide usage practices in the malaria endemic region of North-Western Tanzania: implications to the control of malaria vectors

**DOI:** 10.1186/s12889-019-7767-0

**Published:** 2019-11-06

**Authors:** Anitha Philbert, Sylvester Leonard Lyantagaye, Gamba Nkwengulila

**Affiliations:** 1 Department of Zoology and Wildlife Conservation, University of Dar es Salaam, Dar es Salaam, Tanzania; 2 Department of Molecular Biology and Biotechnology, University of Dar es Salaam, Dar es Salaam, Tanzania

**Keywords:** Pesticides, Insecticides, Malaria vectors, Farmers, Resistance, Magu

## Abstract

**Background:**

Pesticides remain the mainstay for the control of agricultural pests and disease vectors. However, their indiscriminate use in agriculture has led to development of resistance to both crop pests and disease vectors. This threatens to undermine the success gained through the implementation of chemical based vector control programs. We investigated the practices of farmers with regard to pesticide usage in the vegetable growing areas and their impact on susceptibility status of *An. gambiae* s.l.

**Methods:**

A stratified multistage sampling technique using the administrative structure of the Tanzanian districts as sampling frame was used. Wards, villages and then participants with farms where pesticides are applied were purposively recruited at different stages of the process, 100 participants were enrolled in the study. The same villages were used for mosquito larvae sampling from the farms and the surveys were complimented by the entomological study. Larvae were reared in the insectary and the emerging 2–3 days old female adults of *Anopheles gambiae* s.l were subjected to susceptibility test.

**Results:**

Forty eight pesticides of different formulations were used for control of crop and Livestock pests. Pyrethroids were the mostly used class of pesticides (50%) while organophosphates and carbamates were of secondary importance. Over 80% of all farmers applied pesticides in mixed form. Susceptibility test results confirmed high phenotypic resistance among *An. gambiae* populations against DDT and the pyrethroids (Permethrin-0.75%, Cyfluthrin-0.15%, Deltametrin-0.05% and Lambdacyhalothrin-0.05%) with mortality rates 54, 61, 76 and 71%, respectively. Molecular analysis showed *An. arabiensis* as a dominant species (86%) while *An. gambiae s.s* constituted only 6%. The kdr genes were not detected in all of the specimens that survived insecticide exposures.

**Conclusion:**

The study found out that there is a common use of pyrethroids in farms, Livestocks as well as in public health. The study also reports high phenotypic resistance among *An. gambiae* s.l against most of the pyrethroids tested. The preponderance of pyrethroids in agriculture is of public health concern because this is the class of insecticides widely used in vector control programs and this calls for combined integrated pest and vector management (IPVM).

## Background

Malaria is still a major public health challenge globally and especially in Sub-Saharan Africa (SSA) region. According to the 2017 World Malaria report, 91 countries reported 216 million cases of malaria in 2016, this is an increase of 5 million cases compared to the previous records of 2015, the increase in malaria cases has resulted to 445,000 mortalities [1]. Currently, the control of malaria depends on two strategies, which are vector control using insecticides and case treatment using anti-malaria drugs. Nevertheless, vector control is considered as an essential part for reducing malaria transmission [2]. The control of malaria vectors relies on two insecticide based operational scale interventions, indoor residual spraying (IRS) and deployment of long-lasting insecticide-treated nets (LLINs) [2, 3]. The scaling up of insecticide based interventions (IRS and LLINs) has shown positive impacts by reducing both malaria related morbidities and mortalities in various areas such as Equatorial Guinea, Malaysia, Angola and Zanzibar [4–7]. Notwithstanding the achievements gained in the control of malaria through the insecticide based interventions (IRS and LLINs), the challenge is how to sustain the gains. Major challenges are costs, inappropriate use of specific intervention, lack of community acceptance [8, 9] and rising of insecticide resistance in the primary malaria vectors: *Anopheles gambiae* and *Anopheles funestus* species complexes [3].

Magu district, the present study area is one of the districts around the Lake Victoria Zone, the area that has highest malaria prevalence [10]. In attempt to reduce malaria burden in the country, the Government through the National Malaria Control Program (NMCP) has in the past 15 years increased LLINs through universal distribution and increased IRS coverage. In 2006, IRS operations were initiated in Kagera region and by 2011 the operations had extended to most of districts around the Lake Zone area including Magu district. IRS operations started with using a pyrethroid (lambdacyhalothrin), and the insecticides have from time to time been replaced following the emergence of resistance in the targeted vectors. Bendiocarb which is a carbamate was introduced in 2009, and an organophosphate pirimiphos-methyl was recently introduced in in 2014 [10, 11].

Insecticide based interventions are considered the most effective measure to prevent malaria transmission [12]. However, there are a limited number of insecticides that are recommended for use in LLINs and IRS. Currently, most vector control programs are largely dependent on synthetic pyrethroids, which are the only WHO-recommended insecticides for treatment of bed nets [13] and widely used in IRS programs. The same class of insecticide is also used in agriculture and veterinary for control of both crop and livestock pests. Agricultural areas form important breeding habitats of disease vectors [14–16] therefore, such proximity of the vectors with the insecticide residues from both agriculture and public health use poses inherent risk of increased selection pressure to malaria vectors. Globally and in Tanzania, considerable work has been done on human’s health impacts resulting from pesticide misuse and mishandling [17–20], but the effect of these misuse and mishandling to other untargeted species such as disease vectors is under explored. A study by Ngowi et al. [19], reported that about a third of vegetable growers in Northern Tanzania applied pesticides in mixed form. The spill offs from these pesticide cocktails can easily find their way to the mosquito breeding habitats that are located within and around the farms thereby causing selection pressure. These malpractices of pesticide users therefore, warrant further attention to investigate their potential impacts to the environment and other untargeted species such as disease vectors. The present study was therefore designed to assess the practice of farmers with regard to pesticide usage in relation to resistance status of *An. gambiae* s.l in surrounding breeding sites in agricultural settings of Magu district in Tanzania. The indiscriminate use of agricultural pesticides against crop pests constitutes, therefore, a public health issue in most malarious countries.

## Methods

### Study sites

The study was conducted in agricultural areas of Magu district (02°35′S-033°26′E) in Mwanza region, Tanzania. Magu district is bordered to the north by Lake Victoria where most of the vegetables are grown along the Lake Shore. The study was conducted between January through September 2014, and took into consideration the cropping and pesticide application seasons. The farms selected were located in the following vilages: Sola, Ijitu, Kiloleli and Ilumya. The selection of villages was based on the history of past and current use of pesticides (in agriculture, livestock and public health), malaria endemicity and availability of mosquito breeding habitats. The target population was the farmers of vegetables and fruits (tomatoes, cabbages, cucumber and water melons), livestock keepers, dealers of pesticides and other farm inputs in Agroverts, also the agricultural extension officers found within the area.

### Study design

A stratified multistage sampling technique using the administrative structure of the Tanzanian districts as sampling frame was used. Wards, villages and then participants with farms where pesticides are applied were purposively recruited at different stages of the process. The same villages were used for mosquito larvae sampling. Study participants were randomly selected from a list of names of heads of households obtained from the respective village executive officers. A total of 100 participants were enrolled in the study, these were obtained with assistance from the village executive officers and the agricultural extension officers.

### Data collection

Collection of data on the pesticide usage practices was mainly done by two methods: (i) administration of questionnaires (Additional file 1) and (ii) personal observation of the agricultural practices (Pesticide handling, spraying techniques, waste disposal etc) in the farms, shops and households that were visited. To generate adequate information on the practice of farmers, surveys were organized in the study sites of Sola, Ijitu, Kiloleli and Ilumya villages. Structured and semi-structured questionnaires were administered to 100 respondents, the population study composed of farmers, livestock keepers, retailers and dealers of pesticides in agroverts located within the study site. The tool was formulated in English but was translated and administered in Kiswahili (language common to all Tanzanians) to ensure maximum understanding of the questions by the participants but care was taken to retain the original meaning. The questionnaires focused on the history of vegetable farms, the size of farms, educational level of farm owner, type of pesticides used, farmer’s source of information on pesticide use, pesticide sources and availability, farming techniques and the pesticide treatment strategies (spraying methods, mixture and quantities), the frequencies, longevity and time of pesticide application in the farms. Qualitative data were recorded by direct observations and in-depth interview to key informants (extension officers, farmers and pesticide dealers). Direct observations were made in agroverts, farms and households to examine various pesticide management practices such as pesticide mixing (ratios), spraying techniques, storage, packing and disposal. During observations, farmers were not informed beforehand in order to avoid modifications of their behavior with regard to pesticide handling and to reduce the response bias. All respondents gave a verbal consent to participate in the study and were allowed to withdraw from the study if the need arises.

### Mosquito larvae sampling

Mosquito larvae and pupae were collected from the vegetable farms in Magu district and brought to the insectarium at the National Institute for Medical Research (NIMR), Mwanza center for rearing to adults. The collection was conducted between January to September 2014 covering the three main seasons which coincide with pesticide applications: rainy (262.1 mm-January to March), short rains (18.1 mm-May to June) and dry season (104.4 mm-August to September). The collected larvae were sorted and only Anophelines were reared to adults. The larvae were fed on tetramine fish food (0.02 g dissolved in 500 μl of distilled water for (100–120 larvae) in a plastic white tray (15 × 30 cm) and reared at laboratory conditions of 25–27 °C and 67–79% relative humidity (ICIPE,1997 unpublished). The emerging female adults were morphologically identified using the published keys by Gillies and De Meillon [21] also Gillies and Coetzee [22]. Only the mosquitoes in the *An. gambiae* complex were obtained in sufficient number for susceptibility testing. The sibling species of *An. gambiae* s.l were further confirmed during molecular analyses by PCR after susceptibility testing.

### Susceptibility test

The emerging adult females aged 2–5 days old were subjected to susceptibility tests using insecticide-impregnated papers, as described by the WHO testing protocol [23]. The insecticides tested were the five pyrethroids compounds: permethrin (0.75%), deltamethrin (0.05%), lambdacyhalothrin (0.05%), etofenprox (0.5%) and cyfluthrin (0.15%), and the organochlorine DDT (4%). For each insecticide, 15–20 mosquitoes in each bottle were exposed to insecticide and each experiment had 4 replicates and 1 control. DDT was tested to detect the presence of cross-resistance with the pyrethroids in *Anopheles gambiae* s.l. Mosquitoes were exposed to insecticide-treated papers for 60 min and the knock down times were recorded at the following intervals: (10, 15, 20, 30, 45 and 60 min). Mortalities were recorded 24 h post insecticide exposure and according to the 2013 WHO protocol, when the mortality rate is > 98%, the population is considered fully susceptible; a mortality rate between 97 to 98% means resistance is suspected, and when the mortality rate is < 97%, it means the population is resistant and resistance genes have to be confirmed [23]. Surviving specimens were killed by being frozen at − 20 °C, both the dead and surviving specimens were individually kept in a 1.5 ml eppendorf tube lined with silica gel beads for molecular studies.

### Sibling species identification and *Kdr* genotyping

Mosquito species in the *Anopheles gambiae* s.l were identified by optimizing the conditions and following the procedure of Scott et al. [24], the reaction mixture contained primers based on the species composition available in East Africa. The universal primer was 3’GTGTGC CCCTT CCTC GAT GT 5′; the reverse primers were: 5‘AAGTGTCCTTCTCCATCCTA3’-(*An.arabiesnsis*), 5‘CTGGTTTGGTCGGCACGTTT3’ (*An. gambiae* s.s), 5‘TGACCAACCCACTCCCTTGA3’ (*An. merus*), and 5‘CAGACCAA G ATGGTTAGTAT 3’ (*An. quadrianulatus*). These are the common species available in the area [25]. The identification of *Anopheles gambiae* complex sibling species is based on species-specific single nucleotide polymorphisms (SNPs) in the intergenic spacer region (IGS) [24]. The knock down (*kdr*) resistance gene both western and eastern variants were determined by normal PCR following the published procedure of Martinez-Torres et al. [26] and Ranson et al. [27] respectively.

### Data analysis

Data from the questionnaires were entered and double-checked for quality control in the excel sheet. All responses from the structured and semi-structured questions were then summarized for all participants and analyzed using descriptive statistics including frequency distribution, percentages and means. The susceptibility status of malaria vectors (*An. gambiae* s.l) was evaluated based on the 2013 WHO guidelines [23]. Testing the interactions of resistance status among *An. gambiae* populations for the 5 pyrethroids tested was done using Multiple Analysis of Variance (MANOVA). The software used is GraphPad version 6.01. The time taken for 50 and 95% of all mosquitoes to knock down (knockdown times) was estimated by the Log Probit Model analysis using the time-mortality curves. The software used is Origin Pro 8.5, version 84E, 2010, Origin Lab Corporation. In addition, the resistant ratios were calculated using the KDT50 between the wild mosquitoes and the susceptible laboratory strain (Kisumu).

## Results

### Demographic data

The demographics for the 100 participants are summarized as follows: the average age was 37 years, 75% of all participants were males and 25% were females. For the case of farmers, generally, each farmer had worked on a farm at least for the past 10 years. The majority of participants (80%) had completed standard seven education, a few (17%) completed secondary education and very few (3%) had post-secondary education and attended colleges.

### Pesticides used for the control of pests in the surveyed area

A total of 48 pesticides of different formulations were commonly used in the control of crops and livestock pests in the surveyed area (Table 1). This figure was obtained from the summation of the list given by farmers and extension officers that were matched with the list given by pesticide retailers, as well as direct observations in the field by inspecting and direct reading from the pesticide labels, the duplicates were removed. However, the reported total number could be low compared to the actual number since many farmers could not recall all the names of pesticides they usually apply. Pyrethroids were the mostly used class of pesticides (50%), followed by Organophosphates (15%), Carbamates (11%), Organochlorides (9%), and 15% of the pesticides are from other chemical groups. The classification of the pesticides by chemical group, trade name, active ingredients and status of registration is given in Table 1.

**Table 1:** Types of pesticides commonly applied in vegetable farms and on livestocks as reported by farmers in Magu district, Mwanza region

Pesticide chemical group (% in use)	Trade name	Active ingredient	Status of registration
	Albadip	10% Alphacypermethrin	R
	Agrothrin	Lambdacyhalothrin	R
	Bamethrin	Deltamethrin	R
	Baygon	Imiprothrin + Cyfluthrin	R
	Decis 25 EC	Deltamethrin	R
	Degor	Cypermethrin	NR
Pyrethroids (50%)	Cyclone 505EC	Cypermethrin 10% + Chlorpyrifos	R
	Insectido 5EC	Lambdacyhalothrin	R
	Karate 5EC	50 g/L of Lambdacyhalothrin	R
	Kungfu	50 g/L of Lambdacyhalothrin	R
	Cybadip	Cypermethrin	R
	Duduthrin super	Lambdacyhalothrin	R
	Fenom	Lambdacyhalothrin + Profenofos.	R
	Fenkil 20EC	Fenvalerate 20 g/L	R
	Helarat	Lambdacyahalothrin	R
	Paranex 100 EC	Alphacypermethrin 100 g/L	NR
	Rapid attack	Cypermethrin & Imidacloprid	R
	Shamba powder	Deltamethrin & Fenitrothion	R
	Subachlo	Cypermethrin	R
	Suba Deltamethrin	Deltamethrin	NR
	Ultracya 5EC	Lambdacyhalothrin 5%	R
	Farmerzeb	Mancozerb 80%WP	PR
Carbamates (11%)	Linkmil 72 WP	Metalaxyl 80 kg + Mancozeb	R
	Bamic/Ascomec	Abamectin	R
	Milthane super	Mancozeb	R
Organophosphates (15%)	Insecron 720EC	Prefonofos	NR
	Mocron 720EC	Prefonofos	R
	Prefonos	Endosulfan	NR
	Selecron 720EC	Profenofos	R
	Supercron/Mupacron Prefonofos 500EC	Prefonofos 500EC	R
	Profecron/Banofos	Prefonofos 720E C	R
	Sume	Dimethoate	R
Organochlorides (9%)	Muparaxone	Paraquat 270 g/l	RR
	Thiodan	Endosulfan	NR
	Linkonl	Chlorothelonil 50%	
	Thionex	Endosulfan	PR
	Abanil/Bamic	Abamectin 18 g/L (Avermectin)	R
Others	Agriothrine	Agritone	NR
	Ascomine 2–4-D	2-4D Amine salt (Aryloxyalkanoic acid)	R
	Bamitraz	Amitraz (Amidine)	R
	Kumulus DF	80% Sulphur	R
	Movil	Hexaconozole	R
	Tixfix/Tiktik	Amitraz 12.5% EC (Formamidine)	R
	Glyphon (Glyphosate)	Isopropylamine salt (Phosphanoglycine)	R

*R* Registered for use, *NR* Not registered, *RR* Registered for Restricted use, *PR* Provisional Registration (Registration status as per Tanzania Pesticides Gazette, 2011)

### Classification of pesticides based on the pests they control

**Fig. 1:**
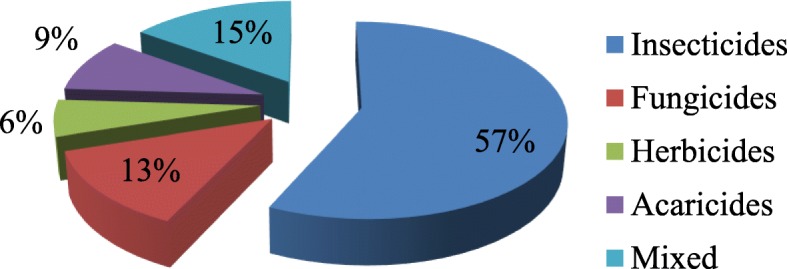
Proportion by class of various pesticides based on the pests they control

The classification of the pesticides based on the type of pests they control revealed that, Insecticides are the most used group (57%), followed by Fungicides (15%), Acaricides (9%), and Hebicides (6%). However, 13% of the pesticides were used multipurposely, for example, a pesticide could be used as an insecticide and acaricide at the same time, example (Duduthrin super- active ingredient: Lambdacyhalothrin). Similarly, some Fungicides (Movil- active ingredient: Hexaconozole) and Herbicides (Agriothrine- active ingredient: Agritone) were also used as insecticides. In Fig. 1, the classification of these pesticides by type of pests they control is presented.

### Pesticide cocktails

During the surveys, tomato farmers were seen mixing various pesticides in the same tanks / container for spraying their crops. Many times, there were no specific mixing instructions or procedures followed. The commonly mixed pesticides were the Fungicides and Insecticides also Fungicide and herbicides as summarized in Table 2:

**Table 2:** Pesticide combinations that were routinely mixed by vegetable farmers in Magu district

Names of pesticides mixed	Pesticide groups	Chemical group
Farm guard + Sume	1Fungicide + 1Insecticide	Pyrethroid + Organophosphate
Shambapowder+ Subachlo + Muparaxone	2Insecticides + Herbicide	2 Pyrethroids + Organochlorine
Farmzeb + Prefenos + Paraquat	1Fungicide+ 1 Insecticide+1Hebicide	Carbamate+ Organophosphate+ Organochlorine
Linkmil + Fenkil + Mo-Karatap+ Farmguard	3Insecticides + 1Hebicide	1Carbamate + 2Pyrethroids

### Pesticide usage practices

The frequency distribution of the responses from farmers on various pesticide usage practices are summarized in Table 3: It was learned from some farmers during the study that they sought information about pesticides because they could not understand the labels, which are normally always written in English, although some were labeled in both languages (Swahili and English).

**Table 3:** Farmer’s various pesticide usage practices as reported by respondents during the survey

Variable	Options	Percentage (%) Response
	Pesticide labels	17.1
	Pesticides retailers/ dealers	45.7
Farmers source of information	Agricultural extension officers	5.7
	Fellow farmers/neighbours	25.7
	News (Tv, radio, magazines)	0
	Others/Unknown	5.7
	**Total**	**100**
	1–5 times per season	10
Pesticide application frequency	5–8 times per season	12.5
	8–12 times per season	42.5
	12–15 times per season	22.5
	More than 15 times per season	5
	Do not know/remember	7.5
	**Total**	**100**
	Environmental pollution	55
Impact of pesticides	Killing of non-targeted species	25
	Pest resistance	12.5
	Others	7.5
	**Total**	**100**
	Pesticides singly applied	20
	Two pesticides mixed	34.28
Pesticide mixing	Three pesticides mixed	28.5
	Four or more pesticides mixed	17.14
	Do not know	0
	**Total**	**100**
	Poured into rivers, lake/ bushes	22.8
Fate of pesticide leftovers	Apply in excess (even when not required)	34.28
	Store for the next application	11.42
	Dispose on the ground/soil	31.42
	**Total**	**100**
Sources of pesticides	Provided by the government	0
	Pesticide retailers shop	85.7
	Others	14.3
	Total	100

### Susceptibility status

A total of 2205 mosquito larvae of *Anopheles gambiae* s.l were collected from which 1500 adults females were obtained. For each season, 450 adults were tested, 75 mosquito adults (60 exposed and 15 control) for each insecticide for the three seasons. The adults were exposed to the Pyrethroids (Permethrin 0.75%, Deltamethrin 0.05%, Lambdacyhalothrin 0.05%, Etofenprox 0.5%, Cyfluthrin 0.15%) and DDT 4%. The susceptibility test results are presented in Table 4. During the season of long rains, the population of *An. gambiae* s.l was fully susceptible to Lambdacyhalothrin (100% mortality) whereas high resistance level were recorded against Cyfluthrin (61% mortality rate) during the same season. High resistance levels were recorded during the dry season against Permethrin, Lambdacyhalothrin and Deltamethrin; mortality rates of 55, 71 and 76% respectively. The mortality rate of *An. gambiae* s.l was consistently less than 5% in all the control groups and therefore the correction factor for the control mortality was not performed. In addition, the reference strain (Kisumu) was fully susceptible (100% mortality) 24 h post exposure to all the insecticides tested. The susceptibility test results are presented in Table 4.

**Table 4:** Mortality rates of *An. gambiae* s.l. from Magu district 24 h post-exposure to pyrethroids and DDT, January–September, 2014

	Long rains season			Dry season			Short rains season		
Insecticide	No of mosquitoes exposed	No of mosquitoes survived	% Mortality ± SD	No of mosquitoes exposed	No of mosquitoes survived	% Mortality ± SD	No of mosquitoes exposed	No of mosquitoes survived	% Mortality ± SD
Kisumu	100	0	100 ± 0	100	0	100 ± 0	60	100	100 ± 0
Permethrin 0.75%	60	9	85 ± 0.063	60	27	54.75 ± 19.29	60	2	97 ± 0.05
Deltamethrin 0.05%	60	7	88 ± 0.062	60	14	76.25 ± 15.756	60	2	97 ± 0.05
Lambdacyhalothrin 0.05%	60	0	100 ± 0	60	17	71.25 ± 14.773	60	0	100 ± 0
Etofenprox 0.5%	60	4	95 ± 0.035	60	0	100 ± 0	60	2	100 ± 0
Cyfluthrin 0.15%	60	23	61 ± 0.115	60	7	88 ± 11.51	60	4	97 ± 0.035
DDT 4%	60	3	96 ± 0.04	60	0	100 ± 0	60	9	84 ± 0.021
Total	360	46		360	65		360	19	

SD is standard deviation: Kisumu is the susceptible laboratory strain used for reference

Moreover, data analysis indicates that mortality rates of *Anopheles gambiae* s.l were significantly influenced by seasons; *p*-value = 0.007, X-squared = 24.421, df = 10 (Pearson’s Chi-squared test). Nevertheless, there was no significant difference of mortality rate between individual insecticides and season, as demonstrated by Multiple Analysis of Variance-MANOVA, (*P* = 0.225, F (1.6, 17)).

### Sibling species identification and *Kdr* genotyping

A total of 130 mosquitoes survived insecticide exposure (Table 4). A total of 1000 mosquitoes were randomly selected from the samples that died after insecticide exposure and 130 specimens that survived the exposure were further subjected to *An. gambiae* s.l sibling species identification by PCR. Out of 1130 specimens, 86% were *Anopheles arabiensis* and only 8% were *Anopheles gambiae* s.s and the remaining 6% of the sampled failed to amplify and couldn’t be identified.

### Knockdown times

The time taken to knock down 50 and 95% (KdT50 and KdT95) respectively are presented in Table 5. The knockdown times were compared against mortality rates of *An. gambiae* s.l. During the short rain season, the knock down times against Lambdacyhalothrin were significantly longer for *An. gambiae* s.l compared to Kisumu; *P* < 0.05.The corresponding values are 28 min, 68 min and 114 min for Kisumu, KdT50 and KdT95 for *An. gambie* s.l respectively and the corresponding resistance ratio was 2.4 implying *kdr* based mechanism of resistance. During the dry season, long knock down times were recorded against Deltamethrin and DDT as compared to Kisumu; P < 0.05 and the corresponding resistance ratios were 2.2 and 1.9 respectively.

**Table 5:** Knockdown times (KdT50 and KdT95) in minutes for *Anopheles gambiae* s.l. populations from Magu agro-ecosystem zone, Mwanza region, January–September, 2014

Season	Insecticide	KdT_50_ Kisumu	KdT_50_ *An. gambiae* s.l.	KdT_95_ *An. gambiae* s.l.	KdT_50_R
	Permethrin 0.75%	44.74 [41–46]	38.01 [31–43]	63. 37 [51–74]	0.85
Long rains	Deltamethrin 0.05%	20.38 [17–22]	28.31 [25–30]	52.67 [51–54]	1.39
	Lambdacyhalothrin 0.05%	28.56 [28–29]	26.05 [19–31]	53.59 [49–60]	0.91
	Etofenprox 0.5%	27.00 [25–28]	43.47 [40–44]	69.51 [64–72]	1.61
	Cyfluthrin 0.15%	16.23 [12–19]	41.98 [39–45]	68.67 [62–77]	2.58
	DDT 4%	35.14 [33–35]	35.49 [34–35]	54.49 [52–57]	1.01
	Permethrin 0.75%	44.74 [41–46]	44.40 [36–68]	72.61 [54–118]	0.99
Dry season	Deltamethrin 0.05%	20.38 [17–22]	45.36 [42–53]	72.31 [63–84]	2.23
	Lambdacyhalothrin 0.05%	28.56 [28–29]	44.85 [40–50]	70.40 [66–80]	1.57
	Etofenprox 0.5%	27.00 [25–28]	34.46 [26–38]	58.22 [52–63]	1.27
	Cyfluthrin 0.15%	16.23 [12–19]	27.91 [24–30]	51.01 [49–53]	1.72
	DDT 4%	35.14 [33–35]	68.11 [45–99]	114.90 [70–180]	1.94
	Permethrin 0.75%	44.74 [41–46]	62.40 [53–69]	103.16 [88–114]	1.39
Short rains	Deltamethrin 0.05%	20.38 [17–22]	40.19 [33–42]	64.29 [56–68]	1.97
	Lambdacyhalothrin 0.05%	28.56 [28–29]	68.82 [58–82]	114.97 [95–139]	2.41
	Etofenprox 0.5%	27.00 [25–28]	48.78 [40–54]	76.09 [62–86]	1.81
	Cyfluthrin 0.15%	16.23 [12–19]	50.25 [46–55]	82.66 [77–92]	3.09
	DDT 4%	35.14 [33–35]	40.18 [38–14]	61.47 [58–64]	1.14

KdT50 refers to time taken to knock down 50% of tested mosquitoes. KdT50R is the resistance ratio (RR) obtained by dividing the

KdT50 of the tested wild population by the KdT50 of the susceptible strain (Kisumu)

### Knock-down resistance mutations (*kdr*)

A total of 130 mosquitoes that survived insecticide exposure were further subjected for *kdr* genotyping (both eastern and western variants). *Kdr* mutation alleles were not detected in all the samples that were tested and therefore sequencing was not performed. This implies that mechanisms other than *kdr* were responsible for the phenotypic resistance recorded among the population of *An. gambiae* s.l in this study site.

## Discussion

The present study has observed the indiscriminate and intensified pesticide use of 48 different formulations in the study area. The use of a high number of different formulations in Tanzania had been reported before. Ngowi et al. [19] reported a total of 40 chemical formulations used by the vegetable farmers in Northern Tanzania. This situation is not unique to Tanzania but also true in many other countries where chemical control of crop pests is a dominant strategy [17, 18, 28]. In Benin, Ethiopia, Ghana and Senegal, Williamson et al. [28] recorded 47 different formulations that were used by farmers to control crop pests. Similarly, Ntow et al. [17] reported 43 pesticides that were used by farmers in Ghana to control vegetable pests.

The proportion by class of pesticides used in the current study is presented in Table 1. Contrary to what have been reported from other agro- ecosystem zones where the organophosphates were the dominant group commonly used [17, 29] in the present study, pyrethroids were the most used group. This observation is of public health concern because this is the only class of insecticide recommended for use in the insecticide treated nets (ITNs and LLNs) and widely used in indoor residual spray (IRS) vector control programs. The multiple and prolonged exposure of pests to pesticides have been reported to increase selection to resistance [30–32].

The composition of *An. gambiae* s.l was dominated by *An. Arabiensis* by 86% and *Anopheles gambiae* s.s 8%. The findings are similar to previous studies [33]. It was important to determine species composition because of their ecological and behaviour variations that determines the appropriate methods for their control. These findings are of public health concern because the vector control interventions currently in use (ITNs/LLINs and IRS) target vectors that feed and rest indoors and are less likely to control *An. Arabiensis* because of its exophilic behavior [34].

Susceptibility test results confirmed high phenotypic resistance among *An. gambiae* populations against the pyrethroids such as permethrin, cyfluthrin, deltametrin and lambdacyhalothrin, mortality rates 54, 61, 76 and 71%, respectively. The plausible explanation for the observed high phenotypic resistance of *An. gambiae* s.l against pyrethroids could be the common use of pyrethroids in agriculture (Table 1) and public health (IRS and LLINs). Previous studies conducted in Tanzania [11, 35–38] indicated wide-spread resistance to pyrethroid, DDT and bendiocarb among malaria vectors. The loss of natural susceptibility of malaria vectors to insecticides in these studies was attributed to IRS and LLINs interventions following the national universal net distribution campaigns of 2011 and the School Net Program of 2013. Nevertheless, the practice of farmers with regard to pesticide use and handling in relation to vector resistance has not been well established. Many farmers (70%) used pesticides in mixed form (Table 2) and about 40% had 3 or more pesticides mixed in the same tank (Table 3). Similar type of pesticide mixing for chemical control of crop pests has been reported from Ecuador [39] and in Indonesia [40]. These pesticides have different modes of action and mechanisms for an intended pest, and thus if not carefully used, resistant mosquito species may develop multiple resistance mechanisms as a result of simultaneous multiple exposures of pesticides due to pesticide cocktails. The practice of pesticide mixing may attest for the failure to detect the knock down resistance (*kdr*) gene despite high phenotypic resistance levels recorded against population of *An. gambiae* s.l in the studied area.

The high phenotypic resistance levels observed in this area (Table 4) is attributed to indiscriminate use of pesticides by farmers. In the present study, it was testified by farmers that pesticide cocktails increased the effectiveness of the compound against pests. Farmers also believed that mixing saved time of spray that could have been spent by spraying one pesticide after another. Surprisingly, mosquitoes and other aquatic invertebrates survived such mixtures of pesticide exposures in the surveyed farms in their immature stages. This high tolerance of mosquito larvae to a mixture of pesticides is of public health concern and warrants further research to examine the mechanisms of vector resistance to multiple exposures of pesticides / insecticides. It was also observed during the survey that famers rinsed their sprayers and mixing containers in water bodies found around and within the farms. Other pesticide wastes and remains were directly discharged within these water bodies. These water bodies form mosquitoes’ breeding habitats and therefore this practice of discharging the pesticide wastes directly in aquatic habitats may increase the selective pressure of mosquito vectors against the relevant insecticides. The same practice has been observed in Nigeria [18] and among the cotton farmers in Cote d’Ivoire [41]. In Cote d’Ivore (ibid), the practice was associated to fish poisoning, whereas, in other places the contamination of aquatic habitat by pesticides has been considered as the main cause of selection for resistance among disease vectors [42–44]. Similar findings were reported from previous study by Nkya et al. [45]. The buildup of resistance gradually can be an irreversible process that will demand use of increased quantities of pesticides, this will not only increase the pesticide production and expenditure costs but also the potential hazardous effects of pesticides to the environment. Further to note, many farmers (70%) used pesticides in mixed form (Table 2) and about 40% had 3 or more pesticides mixed in the same tank (Table 3). Similar type of pesticide mixing for chemical control of crop pests has been reported from Ecuador [39] and in Indonesia [40]. Pesticides mixing have been reported to induce resistance selection pressure among crop pests [46].

Spraying was also done unnecessarily even when there was no infestation and majority of farmers (over 60%) sprayed their farms between 8 and 15 times per cropping season and a few (5%) reported to have sprayed their farms more than 15 times per cropping season. All these practices were contrary to the instruction labels or the information given by the extension officers. It was further narrated that they applied pesticides many times and in large quantities than the recommended dose because small doses were no longer able to kill the pests. There is, therefore, urgent need to address this rampant and indiscriminate use of pesticides in the present study area, and most probably throughout the country where pesticides are intensively used in a similar manner.

Pesticides were readily available in the study area. The major source of pesticides was the agricultural retailer shops that are located a walking distance from farmers’ residencies. It was also observed that pesticide retailers repackaged pesticides into small containers that were cheap and could be afforded even by poorer farmers. The small packs contained no labels about how to use a particular pesticide. In addition, majority of farmers (over 80%) reported retailers or fellow farmers (Table 3) as their source of information. It is, therefore, most likely that farmers receive pesticide information from a third part (fellow farmers), which is not reliable. Pesticide labeling was not consistent, some pesticides had no labels at all, and some were completely labeled in English, a foreign language to the majority Tanzanians and therefore farmers could not decipher the technical knowledge in the labels.

Farmers reported the use of pesticides that are not registered for use during the survey. Pesticides of Endosulfan nature Thionex and Thiodan were freely available for sale in open markets and were being used against tomato pests (Table 1). Participants in the present study, further reported the use of expired pesticides from the previous seasons by mixing them with little amount of new pesticides to reduce the costs. It is, therefore, very likely that pesticide induced effect to humans, and environment and other untargeted species such as vector resistance will continue to increase in Tanzania if appropriate measures will not be instituted in time.

## Conclusion

Generally, the intensive use of pesticides is seen to be triggered by occurrence of serious crop pests, the risk of losing crop yields, pesticides’ / insecticides’ decreased efficacy, farmers desire to increase crop yields and the readily availability of agrochemicals. The high phenotypic resistance levels against Pyrethroids which are not matched with resistance gene imply that vectors have selected for mechanisms other than *kdr* and this calls for further investigations. Furthermore, the lack of extension services and ineffective environmental and pesticide control policies further explains the indiscriminate use of pesticides. The study recommends regular awareness training to farmers on good farming practices and adoption of integrated pest and vector management that will reduce the use of pesticides, and especially use of pyrethroids to reduce the multiple exposure of pests and disease vectors to pesticides. Furthermore, the Government through the respective authorities should expand the extension services to the village level. The strict enforcement of the existing pesticide and environmental laws is also advocated to reduce the extent of environmental contamination and other risks associated with the use of pesticides. This will reduce the spread of resistance in disease vectors, and this can only be achieved through collaboration of the relevant sectors such as agriculture, livestock and vector control units during the planning of any intervention.

## Supplementary information

**Additional file 1.** Questionnaire.

**Additional file 2.** Susceptibility test results rain season Magu.

**Additional file 3.** Susceptibility test results dry season Magu.

**Additional file 4.** Susceptibility test results short rain season Magu.

## Data Availability

All data generated during this study are included in this published article; and its Additional files 2, 3 and 4.

## Abbreviations

CARTA: Consortium for Advanced Research Training in Africa
DDT: Dichlorodiphenyltrichloroethane
IGP: Intergenic Spacer Region
IRS: Indoor Residual Spray
ITNs: Insecticide Treated Nets
KDR: Knock down resistance
LLINs: Long Lasting Insecticidal Nets
MANOVA: Multiple Analysis of Variance
NIMR: National Institute for Medical Research
NMCP: National Malaria Control Program
PCR: Polymerized Chain Reaction
SNP: Single Nucleotide Polymorphisms
SSA: Sub Saharan Africa
