# Supplementary material for: Farmers’ pesticide usage practices in the malaria endemic region of North-Western Tanzania: implications to the control of malaria vectors
Source: BMC Public Health. 2019 Nov 6;19:1456. doi: 10.1186/s12889-019-7767-0 (PMC6833290; doi:10.1186/s12889-019-7767-0)
Supplement: Supplementary file 1 — Additional file 1. Questionnaire. [file 12889_2019_7767_MOESM1_ESM.docx]

**Appendix 2: Questionnaire for the Pesticide stakeholders**

**University of Dar es Salaam Ph.D. Candidate research tool**

**General instructions**

This study is aimed at collecting information on the history of pesticide use for the purpose of revealing environmental effects associated with use of pesticides. The study is exclusively for academic purposes and there is no direct benefit to any participant. However knowledge gained through this study will help plan of proper use of pesticides thereby maintaining the environment and associated ecological habitats of various species through planning of the appropriate interventions. The survey will involve farmers, agricultural extensions officers, community/ political leaders, livestock keepers and businessmen (agrovet shops). For closed ended questions you are requested to choose appropriate answers (or tick) in the space provided and for open ended questions you are requested to give appropriate details.

**Definition of key terms**

**Pesticides** are chemicals that are applied on crops and plants to control pests.

**Pests** are insects that destroy plants or food crops in the farms or stored grains.

**Name of respondent**……………… **Village** ……………..**District**………………

1. Age of respondent………………….. 2. Sex of respondent…………………………………….

3. What is your highest level of education (Choose only one that best describes you).

( ) Primary education ( ) Secondary education

( ) Post-secondary education ( ) Never have been to school

4. What is your occupation (Choose only one that best describes you)

( ) Farmer ( ) Livestock keeper

( ) Mixed (crop and Livestock) ( ) Others (specify)…………………………..

5. What is the size of the farm (in acres) do you own for each crop cultivated: ……………………………….

6. Have you ever used pesticides? (tick the appropriate) ( ) Yes ( ) No

7. If yes, how long have you been using pesticides? (Choose only one that best describes you).

( ) More than 10 ( ) 5-10 years ( ) Less than 5 years

( ) Recently, within a year ( ) Never used ( ) I don’t remember

7. What are the pesticides normally used by the household to control crop pests?

Trade name/local name Active ingredient

1. ……………………………………… ………………………………….
2. ……………………………………… ………………………………….
3. ……………………………………… ………………………………………
4. ………………………………………… ………………………………………
5. ………………………………………… ………………………………………

8. What are the pesticides normally used by the household to control livestock and household pests?

Trade name/local name Active ingredient

1. ……………………………………… ………………………………….
2. ……………………………………… ………………………………….
3. ……………………………………… ………………………………………
4. ………………………………………… ………………………………………
5. ………………………………………… ………………………………………

9. What are reasons for farmers to use the particular above pesticides?

( ) Cost ( ) Availability

( ) User friendly (Environment and organisms) ( ) Others (specify)……………..

10. Are there any use restrictions or prohibitions regarding the use of any of the pesticide (formulation or the active ingredient?

( ) No

( ) Yes (please specify)

( ) If the pesticide is restricted, ask why does the farmer use it?

10. What was the year a particular pesticide use was terminated?

(i) Name the pesticide…………………………

(ii) Year of its termination………………………….

11. In which season are pesticides commonly applied?

( ) Dry, ( ) Rain ( ) Other (specify)………………………

12. What are the intended use (please tick) Insecticide

( ) Tick control ( ) Fungicide ( ) Herbicide

( ) Rodenticide ( ) Other (specify) ……………

13. Where do you normally obtain pesticides?

( ) Provided by the government ( ) Pesticide retailers shop

( ) Others (specify) ……………………………………

14. Where do you seek for information about pesticide usage?

( ) Pesticide labels ( ) Pesticides retailers/ dealers ( ) Agricultural extension officers

( ) Fellow farmers/neighbours ( ) News (Tv, radio, magazines)

( ) Others(specify)…………………

14. How frequently do you/farmers apply pesticides? (Specify the season)

( ) Single application per season

( ) Multiple application (please specify how many times per year)

15. How do you dispose the pesticide wastes, packing materials and leftovers?

( ) Poured into rivers, lake/ bushes ( ) Apply in excess (even when not required)

( ) Store for the next application ( ) Dispose on the ground/soil

( ) Others (specify)…………………………

16a. Do you use the pesticide according to the formulator’s guidelines? (Choose the most

appropriate one)

( ) Yes ( ) No

16b. If yes, in what ways do you change them? (Choose the most appropriate one)

( a ) Quantity more than the prescriptions

( b ) Quantity more than the prescriptions

( c ) Mix with other pesticide groups

16c. If the answer is C in question number 16b above, then name the

pesticides which you routinely mix…………………………………

17. What do you consider as environmental impacts of pesticide usage?

( ) Environmental pollution ( ) Killing of non-targeted species

( ) Pest resistance ( ) Others (specify) ………………………..

Thank you very much for your participation in the study
